# Prognostic value of functional CT imaging in COVID-ARDS: a two-centre prospective observational study

**DOI:** 10.1186/s12931-025-03232-7

**Published:** 2025-05-09

**Authors:** Mehdi Shekarnabi, Alicia Guillien, Nicolas Terzi, Florian Sigaud, Laurent Bitker, Emmanuel Roux, Touria Ahaouari, Eduardo Enrique Dávila Serrano, Loic Boussel, Gilbert Ferretti, Hodane Yonis, Mehdi Mezidi, Ines Noirot, Louis Chauvelot, François Dhelft, Maxime Gaillet, Valérie Siroux, Maciej Orkisz, Jean-Christophe Richard, Sam Bayat

**Affiliations:** 1 Synchrotron Radiation for Biomedicine Laboratory (STROBE), INSERM UA07, Univ. Grenoble Alpes, Grenoble, France; 2 INSA-Lyon, CNRS, INSERM, CREATIS UMR 5220, U1294, Univ. Lyon, Université Claude Bernard Lyon 1, Villeurbanne, U1294 France; 3 Team of environmental epidemiology applied to development and respiratory health, Institute for Advanced Biosciences, Univ Grenoble Alpes, Inserm U1209, CNRS, Grenoble, France; 4 Service de Médecine Intensive Réanimation, CHU Grenoble-Alpes, Grenoble, France; 5 Service de Médecine Intensive Réanimation, Hôpital de la Croix Rousse, Hospices Civils de Lyon, Lyon, France; 6 Service de Radiologie, Hôpital de la Croix Rousse, Hospices Civils de Lyon, Lyon, France; 7 Service de Radiologie Diagnostique Et Interventionnelle, Université Grenoble Alpes, CHU Grenoble-Alpes, Grenoble, France; 8 Université Claude Bernard Lyon 1, Villeurbanne, France; 9 Department of Pulmonology & Physiology, Grenoble University Hospital, Grenoble, France

**Keywords:** Acute respiratory distress syndrome, SARS-CoV-2, Computed tomography, Image registration, lung functional imaging

## Abstract

**Background:**

Patients with ARDS have heterogeneous lungs which exposes them to the risk of lung injury exacerbation by mechanical ventilation. Functional lung CT imaging gives a comprehensive description of regional lung mechanical behaviour. Here, we investigated whether CT registration-based regional lung function parameters are associated with survival in patients with COVID-ARDS.

**Methods:**

We conducted a two-centre prospective observational study of adult COVID-ARDS patients with an indication for CT within 72 h of onset. Dual volume CT images were aligned using image-registration. Regional lung functional parameters, and their spatial distributions, were analysed by univariable Cox proportional hazard models with survival as the main outcome. Selected variables based on the univariable analysis were included in a stepwise Cox model adjusted for age, sex, body mass index and SAPSII.

**Results:**

94 patients were included in the study. Recruitment was associated with a higher (HR = 1.45, *p* = 0.023) hazard of death, while apical (sΔV_z_) and central (sΔV_x_) displacement of specific volume change centre-of-mass were associated with a lower hazard of death (HR = 0.72, *p* = 0.041; HR = 0.68, *p* = 0.031, respectively).

**Conclusions:**

Our data show that in addition to recruitment, the spatial distribution of specific volume change, a surrogate measure of regional lung ventilation, is associated with the risk of death in mechanically ventilated COVID-19 ARDS patients. Our findings suggest that CT image-registration based functional biomarkers may have prognostic value in COVID-ARDS patients.

**Trial registration:**

This study was retrospectively registered in Clinical Trials under NCT06113276 (https://clinicaltrials.gov/study/NCT06113276) on 27/10/2023.

**Supplementary Information:**

The online version contains supplementary material available at 10.1186/s12931-025-03232-7.

## Introduction

Acute respiratory distress syndrome (ARDS) is a complex and diverse syndrome encompassing a range of clinical and microbiological aetiologies, plasma protein and genomic biomarkers, gas exchange and, importantly, lung mechanical abnormalities [1]. A recent ATS statement recommends advancing data science approaches to facilitate precision medicine strategies for ARDS [2].

Heterogeneous mechanical abnormalities in the lungs are consistently observed in all patients with ARDS [3]. While mechanical ventilation remains the cornerstone of therapy for moderate to severe ARDS cases, it carries the risk of exacerbating lung injury by subjecting mechanically non-uniform lung tissue to excessive stress and strain [4]. The full spectrum of local lung mechanical behaviour in a heterogenous lung is impossible to assess with standard respiratory mechanics.

Advances in computed tomography (CT) image processing allow the assessment of regional lung function based on the precise matching of CT images acquired at 2 different levels of lung inflation [5]. Such image-registration approaches enable us to examine alterations in lung density within spatially matched image voxels as inflation occurs [6], quantifying the regional volume change and its spatial distribution. Functional CT imaging also allows the quantitative assessment of lung regions that are hyperinflated, tidally recruited or completely collapsed. However, the potential prognostic value of functional imaging variables, derived from cutting-edge CT image processing in ARDS remains unexplored.

This study aims to leverage the analysis of regional lung functional parameters, derived from dual-volume CT images, encompassing parameters such as regional lung strain, density change, deformation, recruitment, hyperinflation, and their spatial distributions. We hypothesized that quantitative lung morphological and functional CT imaging parameters or their spatial distribution are associated with clinical outcome, in patients with COVID-ARDS. Some of the raw CT data of this study have been used in previous studies [7, 8].

## Methods

### Study design

This study was a secondary analysis of an ongoing prospective observational multicentre study performed in two intensive care units (ICU) located in university hospitals. The study was approved by an institutional ethics committee (CSE HCL20_194) and registered in Clinical Trials (NCT06113276). Consent for data utilization was sought from the patients or their representatives. More details on methods are provided in the Online Data Supplement.

### Study population

97 eligible patients older than 18 years of age with ARDS [9] under invasive mechanical ventilation, with a ratio of oxygen partial pressure in arterial blood over inspired oxygen fraction (PaO_2_/FiO_2_) below 300 Torr, and an indication for CT according to their attending physician within 72 h of ARDS onset, were enrolled from November 2020 to December 2021. The patients were followed up for 90 days after inclusion. PaO_2_/FiO_2_ and static respiratory compliance (C_rs_, defined as: VT / (P_plat_ – PEEP); where VT: tidal volume; P_plat_: plateau pressure; PEEP: positive end-expiratory pressure) were also recorded for each patient.

### CT imaging and image processing

Low dose CT acquisitions were performed in supine position during end-inspiratory and end-expiratory pauses at 15 (P15) and 5 cmH_2_O (P5) from apex to lung base. The lungs were manually segmented using the CreaTools software [10], excluding pleural, hilar and mediastinal structures (8). The distribution of non-aerated and poorly-aerated regions, defined respectively by density values of: -100–100, and − 500 – -99 HU, was quantified within the apical and caudal halves and left and right lungs of P15 images and expressed as the percentage of total lung volume.

To measure regional lung deformation with lung inflation, the Dense Displacement Sampling (DEEDS) algorithm for non-rigid registration of P5 and P15 images was used [11]. Image-based outcomes were computed in 4 × 4 × 4 voxel regions in order to minimize potential errors due to voxel misalignment. The following metrics were extracted from the registered images: lung attenuation change (ΔHU); gas volume change (ΔVg); Jacobian determinant (J; *J > 1 or J < 1*, indicate local expansion or contraction, respectively); regional lung recruitment (Rec); and hyperinflation (HI). Specific regional gas volume change (sΔV) was defined as ΔV normalized to the regional gas volume at P5. For each metric, descriptors of the statistical (median, IQR, skewness, kurtosis) and spatial distribution (relative distance of the centre of mass from centre of the normalized image grid), were computed. Furthermore, an average map was computed for all patients in both the survivor and non-survivor groups for each metric. A detailed description of all variables is provided in supplemental Tables 1 & 2.

### Statistical analysis

Non-normally distributed continuous variables were transformed by the Yeo-Johson power transformation [12]. Each variable was centred by its mean and rescaled by its standard deviation to normalize effect size across all variables. No missing data were imputed. Subject characteristics and image-derived variables were compared between survivors and non-survivors using Fisher’s exact and Kruskal-Wallis tests for discrete and continuous variables, respectively. Correlation was assessed by the Pearson correlation coefficient. The association of image registration-derived variables and survival was assessed by univariate Cox proportional hazards analysis. Variables with a *p*-value ≤ 0.1 were entered into a Cox proportional hazards model, using a variance inflation factor ≤ 2 to detect multicollinearity. Stepwise model selection based on the Akaike Information Criterion (AIC) was performed to simplify the model. The final Cox model included the selected imaging variables along with clinical covariates such as age, sex, body max index (BMI), and the simplified acute physiology score (SAPSII) computed without age contribution. The pre-processing and analysis workflow was implemented using R version 4.3.2 [13], with dplyr, bestNormalize, survival, and survminer packages. A *p*-value < 0.05 was considered significant.

## Results

### Study cohort

Out of 99 initially enrolled subjects, 5 subjects were excluded from the analysis; one had missing clinical data, one due to a large pleural effusion, 3 had negative polymerase chain reaction (PCR) tests. 94 patients were included in the final analysis. Characteristics of the subjects are summarized in Table 1. Non-survivors had a significantly higher SAPSII score and lower PaO_2_/FiO_2_. Mechanical ventilation settings, ECMO prevalence, age, BMI and respiratory compliance were not significantly different between survivors and non-survivors.

**Table 1:** Characteristics of the studied population included within each cluster

Characteristic	All Subjects	Non survivors	Survivors	*p*-value
*n* [%]	94	46 [47%]	48 [50%]	
Female, *n* [%]	24 [26%]	12 [26%]	12 [25%]	n.s.
Age, years	61 [53, 68]	63 [53, 70]	60 [51, 65]	n.s.
BMI	31 [27–36]	31 [26–38]	31 [28–35]	n.s.
SAPSII	39 [30, 45]	41 [34, 47]	33 [29, 42]	0.011
Vasopressor, *n* [%]	69 [71%]	31 [67%]	35 [73%]	n.s.
VT at inclusion, ml·kg^-1^ PBW	3.50 [1.97, 4.70]	3.31 [0.94, 4.61]	3.98 [2.67, 4.73]	n.s.
PEEP, cmH_2_O	10 [5, 14]	10 [8, 15]	10 [5, 13]	n.s.
PEEP_tot, rs,_ cmH_2_O	10 [7, 15]	11 [8, 15]	10 [7, 14]	n.s.
P_plat, rs_, cmH_2_O	20 [17, 22]	20 [17, 22]	21 [17, 23]	n.s.
ΔP_rs_, cmH_2_O	9 [7, 12]	9 [6, 11]	10 [8, 12]	n.s.
PaO_2_/FIO_2_ *	85 [70–108]	78 [65–91]	88 [74–123]	0.014
pH	7.38 [7.32, 7.45]	7.38 [7.30, 7.45]	7.38 [7.36, 7.45]	n.s.
ECMO	24 (26%)	13 (28%)	11 (23%)	0.6
VFD m [SD]	31 [35]	0 [0]	60 [25]	< 0.001
C_rs_	33.7 [25.0–45.3]	31.8 [24.8–46.3]	35.2 [25.9–44.7]	0.5

Data are median [1st quartile, 3rd quartile] unless otherwise stated. SAPSII: Simplified Acute Physiology Score II; VT: tidal volume; PBW: predicted body weight; PEEP: positive end-expiratory pressure at inclusion; PEEP_tot, rs_: positive end-expiratory pressure of the respiratory system at inclusion; P_plat, rs_: plateau pressure of the respiratory system at inclusion; ΔP_rs_: driving pressure of the respiratory system at inclusion; PaO_2_/FIO_2_: arterial O_2_ pressure/inspired O_2_ fraction; ECMO: extracorporeal membrane oxygenation; C_rs_: static respiratory compliance; VFD: ventilator-free days. *: PaO_2_/FmO_2_ for patients under ECMO where FmO_2_ is the membrane O_2_ fraction; n.s.: no statistically significant difference

### Regional distribution of lung density

The distribution of non-aerated and poorly-aerated tissue fraction within the apical and caudal halves, and within the right and left lung are summarized in Table 2. A significantly larger fraction of non-aerated regions was observed in caudal regions irrespective of survival status. The non-aerated fraction was not significantly different between the right and left lungs. There were no significant differences in the non-aerated tissue fraction between survivors and non-survivors. Regarding poor aeration, there was no significant difference between apical and caudal regions. The poorly aerated fraction was however significantly larger in the left lungs in both survivors and non-survivors. Moreover, the left lungs of non-survivors had a significantly higher fraction of poor aeration compared to survivors (10.0 [6.5–12.8] vs. 7.8 [6.2–9.6], *p* = 0.044).

**Table 2:** Distribution of non-aerated and poorly-aerated tissue fraction

Tissue fraction (%)		Non-Survivor	Survivor	
Non-Aerated				*p*-value
	Apical	8.1 [4.5–16.7]	6.6 [3.3–15.2]	n.s.
	Caudal	15.7 [10.3–22.3]	17.2 [10.5–28.4]	n.s.
	*p*-value	< 0.0001	< 0.0001	
	Right	10.9 [8.0–18.4]	10.9 [7.1–19.7]	n.s.
	Left	12.7 [7.1–20.9]	12.2 [7.5–26.3]	n.s.
	*p*-value	n.s.	0.021	
Poorly-Aerated				
	Apical	10.1 [5.6–11.7]	7.1 [5.2–9.4]	n.s.
	Caudal	9.1 [5.7–11.6]	7.5 [4.9–10.7]	n.s.
	*p*-value	n.s.	n.s.	
	Right	8.6 [4.9–11.2]	6.3 [4.8–9.3]	n.s.
	Left	10.0 [6.5–12.8]	7.8 [6.2–9.6]	0.044
	*p*-value	< 0.0001	0.014	

The percentage of non-aerated [-100–100 HU] and poorly-aerated [-500 – -99 HU] lung tissue within apical, caudal, right and left lungs at 15 cmH_2_O. Data are median [1st quartile – 3rd quartile]; n.s.: no statistically significant difference

### Regional lung functional variables associated with higher mortality

Table 3 defines the image-derived functional variables included in the final Cox proportional hazards model, and summarizes their median values in survivors and non-survivors. In this model adjusted on age, sex, BMI and SAPSII, the global recruited lung tissue volume (V_rec_) was associated with a higher hazard of death (Table 4). Conversely, a regional specific volume change skewed apically (sΔV_z_ increase) and towards the centre (sΔV_x_ increase), was associated with a reduced hazard of death.

**Table 3:** Definition and median value of image-derived variables included in the Cox proportional hazards regression model

Variable	Definition	Unit	All Subjects	Non survivors	Survivors	*p*-value
V_rec_	Global recruited lung tissue volume 5 → 15 cmH_2_O	L	0.11 [0.07–0.19]	0.14 [0.09–0.23]	0.09 [0.07–0.15]	0.028
sΔV z	Specific volume change, centre of mass z relative distance from grid origin	%	15 [8, 23]	10 [6, 22]	17 [10, 24]	0.050
sΔV x	Specific volume change, centre of mass x relative distance from grid origin	%	-3 [-11, 3]	-5 [-15, 0]	-1 [-7, 4]	0.10

Data are median [1st quartile – 3rd quartile]. See online supplement (Supplemental Tables 1 & 2) for detailed computation of each image-based variable; *p*.-value compare survivors to non-survivors

**Table 4:** Stepwise Cox proportional hazards regression

Predictor	HR	95% CI	*p*-Value
sΔVz	0.72	0.53, 0.98	0.041
V_rec_	1.45	1.05, 2.00	0.023
sΔVx	0.68	0.48, 0.96	0.031
Age	1.14	0.83, 1.56	0.100
Sex (F)	1.48	0.75, 2.92	0.200
BMI	1.12	0.76, 1.66	0.600
SAPSII	1.48	1.11, 1.97	0.010

Stepwise Cox proportional hazards regression model, adjusted on age, sex, BMI and SAPSII score without age contribution. HR: hazard ratio; CI: confidence interval. Image-derived variables are defined in supplemental Tables 1 & 2. SEX (F): female sex

**Fig. 1:**
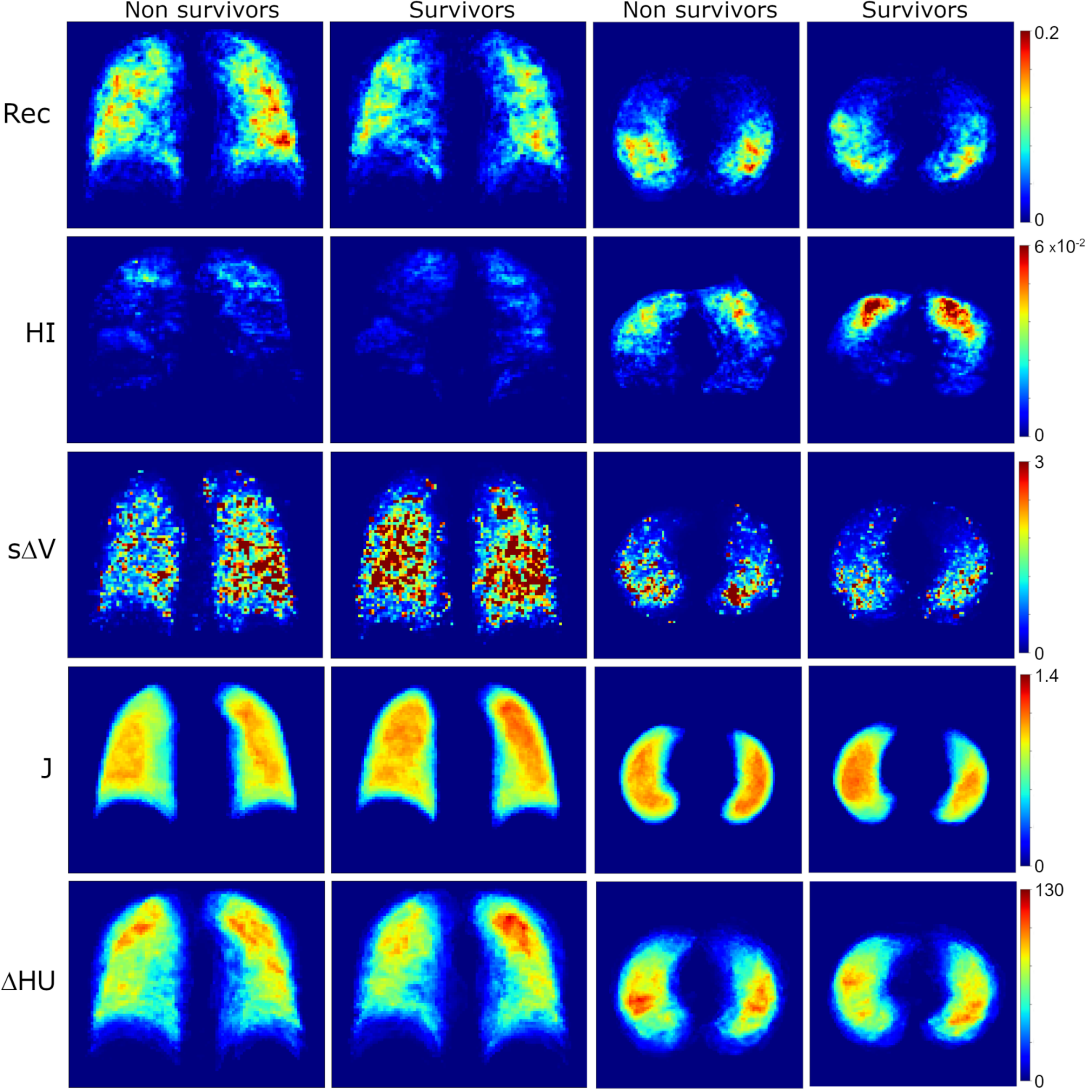
Average distribution of CT-derived functional parameters on a normalized image grid as a function of survival status at 90 days. Rec: recruitment (fraction of tissue mass per region); HI: hyperinflation (fraction of tissue mass per region); sΔV: specific volume change from 5 to 15 cmH_2_O (unitless). ΔHU: density change from 5 to 15 cmH_2_O. J: Jacobian determinant. Note the increased recruitment, a less left-skewed and more apically distributed sΔV in survivors

Figure 1 shows the spatial distribution of average regional lung function parameters projected on a normalized image grid for all patients within survivor and non-survivor groups. Qualitative analysis suggests that recruitment was larger in non-survivors and more dorsally distributed. Hyperinflation was more pronounced in survivors and predominantly ventrally distributed. Specific volume change, had a more caudal and left skewed centre-of-mass in non-survivors compared to survivors.

**Fig. 2:**
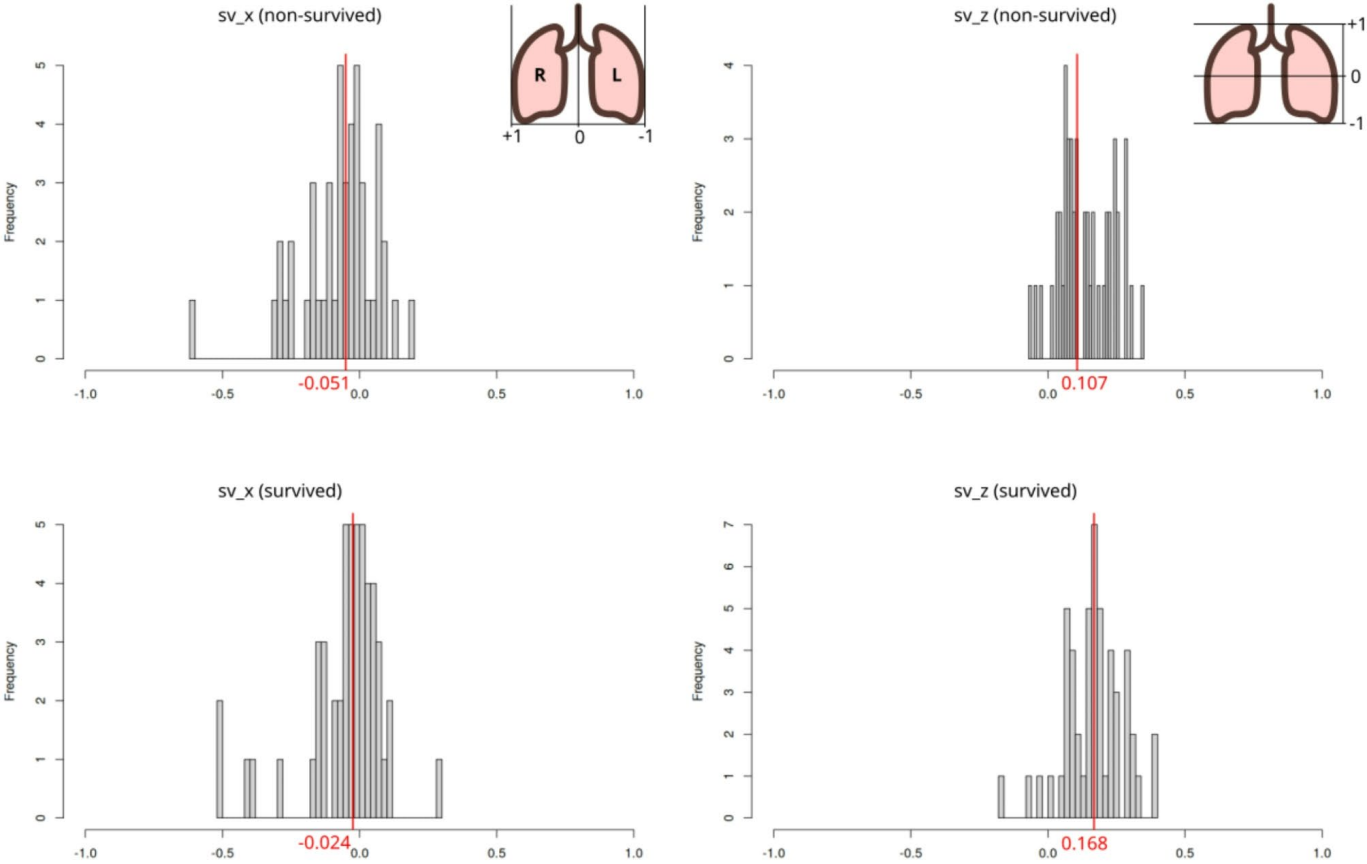
Specific volume change distribution vs. survival status at 90 days. sΔVx: distribution of specific volume change centre-of-mass on the lateral (x) axis. Inset figure shows the direction of change; sΔVz: distribution of specific volume change on the apical-caudal (z) axis. Values vary on a relative scale from − 1 (-100%) to + 1 (+ 100%) with reference to the origin (0) of a normalized grid on which the individual datasets are projected. Red line indicates median value. Note the more caudal and less left-skewed sΔV centre-of-mass in survivors vs. non-survivors

Figure 2 shows the distribution of specific volume change centre of mass along the lateral (x) and apical-caudal (z) axes of a normalized grid on which the data from all subjects within each group are projected. The values are expressed on a relative scale from − 1.0 to + 1.0 and illustrate the values expressed as percentage in Table 3: sΔV distribution leaned apically and was less left-skewed in survivors vs. non-survivors.

Over the entire cohort (both survivors and non-survivors), mean ΔV and Jacobian (J) over the whole lung were positively correlated to C_rs_ (*R* = 0.59, *p* < 0.0001; *R* = 0.49, *p* < 0.0001, respectively). On the other hand, ΔV skewness, a marker of ventilation inhomogeneity, was inversely correlated to both C_rs_ (*R*=-0.56, *p* < 0.0001), and PaO_2_/FIO_2_ (*R*=-0.28, *p* = 0.007). ΔV_x_ and J_x_ were positively correlated to C_rs_ (*R* = 0.28, *p* = 0.007; *R* = 0.28, *p* = 0.007, respectively). Also, both ΔV_x_ and J_x_ were positively correlated to ventilator-free days (*R* = 0.22, *p* = 0.036; *R* = 0.25, *p* = 0.016, respectively). Patients with higher compliance had more hyperinflation (*R* = 0.47, *p* < 0.0001).

**Fig. 3:**
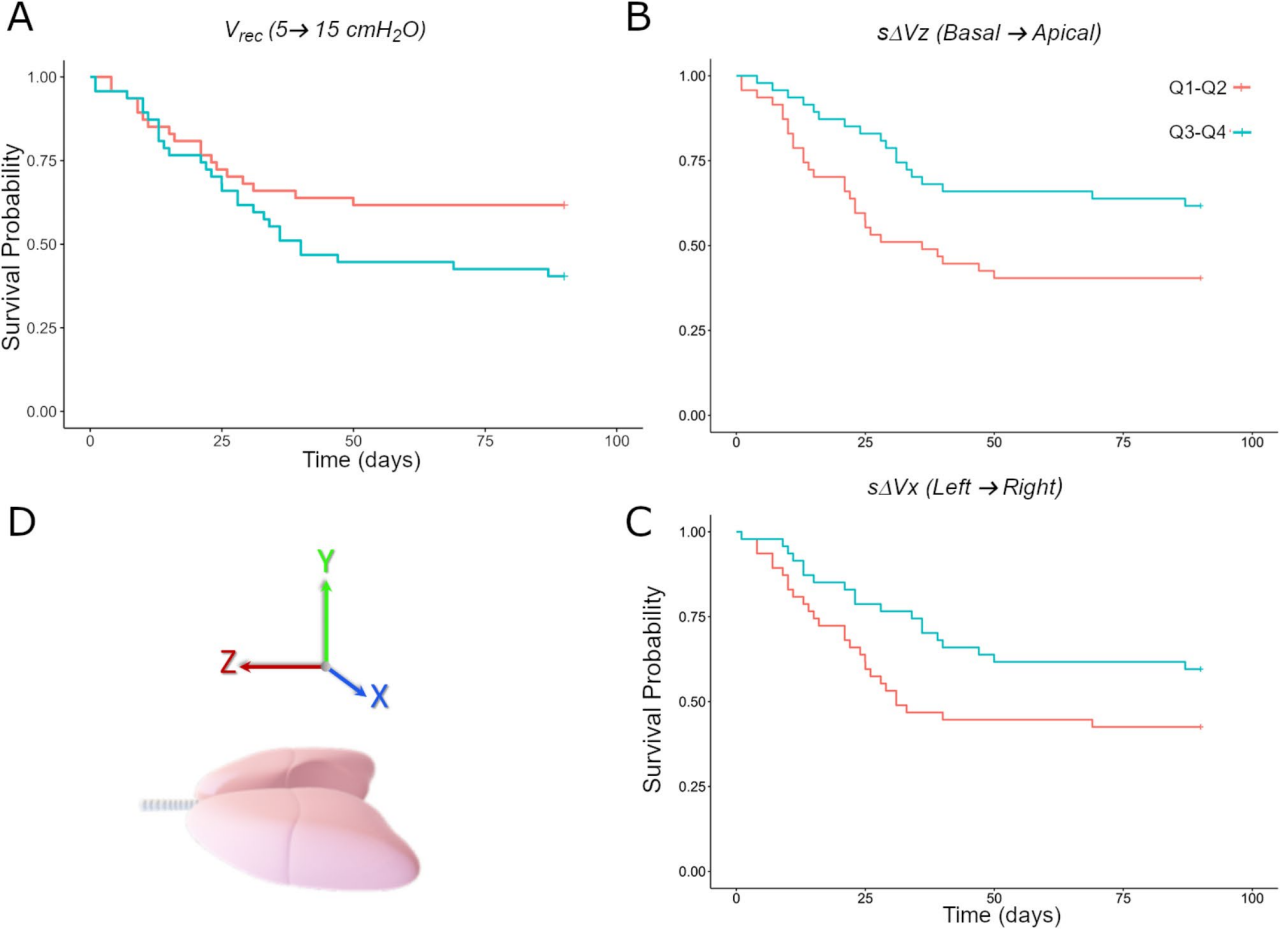
Kaplan-Meier survival probability by lung image-derived variable, comparing: Q1-Q2: 1st and 2nd quartiles; and Q3- Q4; 3rd and 4th quartiles. **A**: V_rec_: Global recruited lung tissue volume between 5 and 15 cmH_2_O; sΔVx: Lung volume change spatial distribution along the left – right axis; sΔVz: Lung volume change spatial distribution along the basal – apical axis. **D**: the sΔV x, y and z axis direction of change and orientation in the supine position

Kaplan-Meier survival probability as a function of time is shown in Fig. 3. The 1st and 2nd quartiles are compared to the 3rd and 4th quartiles, showing a lower survival probability with a higher global V_rec_ between 5 and 15 cmH_2_O, while right (x axis), and apical (z axis) displacement of the centre of mass of sΔV was associated with a lower hazard of death.

## Discussion

The analysis of CT-registration-based metrics investigating regional lung ventilation and biomechanics in moderate to severe COVID-19 ARDS patients undergoing mechanical ventilation revealed that increased recruitment, alongside a more caudal and leftward distribution of lung volume change, were identified as factors associated with elevated risk of death. To our knowledge, this is the first study to assess the spatial distribution of volume change under controlled ventilation and its association with the risk of death in ARDS patients.

Survivors and non-survivors were expectedly distinguished by the severity of ARDS as assessed by the SAPSII score, or PaO_2_/FIO_2_ at PEEP 5 cmH_2_O. However, there were no significant differences in other parameters such as ventilator settings, ECMO prevalence or vasopressor use, that could potentially explain the clinical outcome. The association between a more recruitable lung and increased mortality is well known [14], but is generally thought to be directly correlated to ARDS severity [14, 15]. In this study the higher hazard of death associated with the global recruited volume remained significant after adjusting for severity as assessed by the SAPSII score. Recently, Wendel Garcia et al. retrospectively applied cluster analysis on 54 respiratory mechanics, gas exchange, and CT-derived gas and tissue volume variables to identify a recruitable and a non-recruitable subgroup [16]. They found a higher mortality rate in their recruitable subgroup with a HR of 2.9, independent of SAPSII score, although PaO_2_/FiO_2_ and C_rs_ was lower in this subgroup. The increased hazard of death with increased recruitability in the present study is in line with their findings.

We found that the regional distribution of volume change towards the lung apex is associated with an improved survival. In COVID ARDS, several meta-analyses have demonstrated a predominant distribution of typical ground glass and condensation lesions to the lower lobes [17–20], particularly if the non-aerated caudally-distributed regions are less recruitable. This pattern of distribution however, is not specific to COVID-ARDS, since the loss of aeration and oedema are predominantly present in the lower lobes in all-cause ARDS as well [21]. Also, previous studies have suggested that patients with non-focal ARDS presenting diffuse or patchy loss of aeration, have higher mortality, lower lung compliance and a higher amount of recruitable lung, than patients with focal ARDS [14, 22, 23]. In the present study we also observed a higher fraction of non-aerated lung tissue in the caudal half of the lung (Table 2). However, the lack of differences in aeration loss within apical and caudal halves in survivors and non-survivors may indicate that dividing the lungs into only two regions is too crude an approach to capture meaningful variations in aeration patterns. Together, these findings could suggest that while focal loss of aeration in the lower lobes may redistribute ventilation towards the apices, a pattern of aeration loss that is more diffuse as assessed by CT, could result in a ventilation distribution that is more scattered and less distinctly shifted towards the lung apex.

The association between a leftward shift of regional specific lung volume change (sΔVx) centre-of-mass and a higher hazard of death is more intriguing. Part of the leftward shift may be due to the anatomic disposition of the mediastinum which may skew the sΔVx leftwards. On the other hand, the pattern of parenchymal aeration distribution within the lung may be involved. While some meta-analyses suggest a higher prevalence of right lower lobe involvement in COVID-19 pneumonia [17, 24, 25], others do not confirm this result [18, 20]. We found no difference in the fraction of non-aerated tissue between the left and right lung in this study, although there was a significantly higher fraction of poorly aerated parenchyma in the left lung. Poor aeration could have shifted sΔV in subjects at risk of a poor outcome, given that this parameter is normalized to the regional gas volume (see: online supplement). Again, comparing left and right lungs may be too crude to detect the fine distribution of sΔV.

The potential clinical implication of our study is that image registration-based lung functional variables may be of value to inform and enrich a personalized mechanical ventilation approach or patient selection for future clinical trials. One example is identifying which patients may benefit from higher PEEP settings, within the limits of cardiovascular tolerance. This paradigm has been shown to be challenging in the past. Previously, the LIVE randomized controlled trial aimed at personalizing mechanical ventilation based on lung CT morphology [26]. Personalized mechanical ventilator settings including recruitment manoeuvres and higher PEEP were proposed in non-focal vs. focal ARDS in the treatment arm, the latter subgroup having been found to be more recruitable [23]. In an intention-to-treat analysis, there was no difference in 90-day survival, however, 21% percent of the patients were misclassified. Although there was a benefit of personalized ventilation in the appropriately classified patients, mortality was higher in the misclassified subjects. Unlike this approach, where lung morphology was qualitatively assessed by an investigator, in the present study the measurement of image-based lung function parameters uses objective measurements, through fully automatized, and operator-independent assessment of lung CT images.

Our study had strengths and limitations. Our data are limited to a category of patients with COVID pulmonary ARDS. Although early on, COVID ARDS was thought to have specific respiratory mechanical features with higher compliance in some patients despite profound hypoxemia [27, 28], subsequent data in larger cohorts have challenged this view based on the finding that in COVID-ARDS the lung is not mechanically different than non-COVID [29–31]. Nevertheless, we cannot exclude patterns of loss of aeration that are associated with COVID-19. Although the magnitude of the differences in ΔV_x_ and ΔV_z_ appear small, it should be kept in mind that this magnitude depends on the width of the grid upon which the data of the entire patient cohort were projected. Had we applied a narrower grid boundary (see: Fig. 2) the differences in centre of mass location would have appeared larger. Our exploratory study was conducted in a relatively small number of subjects. Therefore, generalization of our findings to all-cause ARDS patients warrants caution. Some potential confounders such as steroid or antiviral drug administration were not included in the analysis. Quantitative CT is the reference method for lung imaging. Transporting patients to the imaging facility is difficult and radiation dose is a concern with repeated CT examination, although low dose CT is increasingly performed with improving image quality [32, 33]. We investigated a limited pressure range on CT imaging, which was dictated by the restricted number of image acquisitions to reduce ionising radiation exposure. This pressure range however, closely reflects the actual tidal expansion. However, the recruitment/derecruitment phenomena are highly non-linear [34] and time-dependent [35, 36] as a function of pressure change. Using only 2 static pressure conditions may underestimate the actual recruitability of the lung tissue. Further study should address the question of functional CT variables with physician-set PEEP and plateau pressures. Finally, clinical implementation of image processing methods based on dual-volume CT imaging is challenging. The data analysis is sophisticated and time-consuming, which impedes its prospective application. However, a fully automated data processing and analysis pipeline is a realistic future perspective.

In conclusion, dual-volume CT-registration based regional lung functional variables, particularly ventilation redistribution towards the lung apex was associated with a significantly lower risk of death at day 90, independent of ARDS severity. These results suggest that CT image-registration based functional biomarkers may have prognostic value in COVID-ARDS patients. Future validation in larger studies may allow assessment of such parameters for personalized mechanical ventilation settings in patients with ARDS.

## Electronic supplementary material

Below is the link to the electronic supplementary material.

Supplementary Material 1

## Data Availability

The datasets used and/or analysed during the current study are available from the corresponding author on reasonable request.
