# Supplementary material for: Prognostic value of functional CT imaging in COVID-ARDS: a two-centre prospective observational study
Source: Respir Res. 2025 May 9;26:177. doi: 10.1186/s12931-025-03232-7 (PMC12065372; doi:10.1186/s12931-025-03232-7)
Supplement: Supplementary file 1 — Supplementary Material 1 [file 12931_2025_3232_MOESM1_ESM.docx]

**Prognostic Value of Functional CT Imaging in Covid-ARDS: a Two-Centre Prospective Observational Study**

**ONLINE SUPPLEMENT**

Mehdi Shekarnabi^1,2^, Alicia Guillien^3^, Nicolas Terzi^4^, Florian Sigaud^4^, Laurent Bitker^1,5^, Emmanuel Roux^1^, Touria Ahaouari^2^, Eduardo Enrique Dávila Serrano^1^, Loic Boussel^1,6^, Gilbert Ferretti^7^, Hodane Yonis^5^, Mehdi Mezidi^5,8^, Ines Noirot^5^, Louis Chauvelot^5^, Francois Dhelft^5,8^, Maxime Gaillet ^5,8^, Valérie Siroux^3^, Maciej Orkisz^1^, Jean-Christophe Richard^1,5^ & Sam Bayat^2,9^

**Corresponding Author:**

Prof. Sam Bayat MD PhD

Department of Pulmonology & Physiology,

Grenoble University Hospital, Bd. Du Maquis du Grésivaudan,

38700, La Tronche, France

email: [sbayat@chu-grenoble.fr](mailto:sbayat@chu-grenoble.fr)

Phone: +33 614 97 17 09

**SUPPLEMENTAL METHODS**

*Image processing*

Interactive segmentation process was carried out by two independent observers using CreaTools software (1). Lung contours were manually selected on a few selected slices, which were then interpolated to generate a 3D segmentation mask. Subsequently, a manual inspection and correction of masks were performed to exclude pleural effusions, main bronchi, and main pulmonary arteries from the segmentation.

A rigid registration of the non-masked images was performed using the Elastix toolbox (2). The P5 image was taken as the moving image, while P15 was used as the fixed image. We chose mutual information as the similarity metric to optimize transformation parameters in a multiresolution setting with a down sampling factor of 2 in four levels using stochastic gradient descent optimizer with 250 iterations per level. We used linear interpolation for resampling between resolution levels and third-order B-spline interpolation to apply the transformation to the moving image. The Dense Displacement Sampling (DEEDS) algorithm for non-rigid registration of P5 and P15 images was then used (3) to warp the P5 image to match the P15 image.

*Computation of regional lung function variables*

Image-based outcomes were computed in 4 × 4 × 4 voxel regions in order to minimize potential errors due to voxel misalignment. All equations are described in (4). A list of the computed variables and their definitions is shown in Supplemental Table 1.

Lung tissue volume at 5 (V_tissue-P5_) and 15 cmH_2_O (V_tissue-P15_) is calculated at the respective pressures as: [(I/1000)+1]×v, where I is the attenuation comprised between -1000 and 0, and v is the region volume (5).

Lung gas volume at 5 (V_g5_) and 15 cmH_2_O (V_g15_) is calculated at the respective pressures as: -(I/1000) ×v. Lung volume at the respective pressures (VL_P5_, VL_P15_) is: V_tissue_ + V_g_.

Regional lung x-ray attenuation change (ΔHU): This metric represents the difference in average CT numbers between corresponding P5 and P15 regions, measured in Hounsfield Units.

Normalized regional gas volume change (ΔV): The gas volume (V) is calculated by multiplying the sum of fractional gas content of voxels in a region (Fgas) by the voxel volume (Vvox). Fgas is calculated from image intensities using Simon’s method (5), which involves dividing the intensity by -1000. ΔV represents the difference in regional gas volume between corresponding P5 and P15 regions. ΔV, it is normalized to the region volume at P5, including tissue and gas.

Specific regional gas volume difference (sΔV): ΔV normalized to the region gas volume at P5.

Regional Jacobian determinant (J): This metric is approximated as the ratio of regional voxel counts of P15 to P5. It corresponds to the Jacobian determinant of the inverse transformation between the 2 volumes.

Normalized regional lung recruitment (Rec): This metric represents the regional difference in tissue mass of non-aerated voxels between P5 and P15 (6). Non-aerated voxels are defined as those with intensities in the range of [-100, 100] HU. The lung tissue density is assumed to have a density of 1 g/ml, and recruitment is expressed in grams. To standardize Rec between patients, it is divided by the initial regional tissue mass and expressed as percentage.

Normalized regional hyperinflation (HI): This metric represents the difference in the count of overinflated voxels between P15 and P5. Overinflated voxels are defined as those with intensities in the range of [-1000, -900) HU at P15, and expressed as the tissue mass of the corresponding voxels in grams. HI is normalized to the P5 tissue mass and expressed as percentage.

For each of the regional parameters: ΔHU, ΔV, J, Rec, and HI, the mean, median, standard deviation (SD), interquartile range (IQR), skewness, and kurtosis was computed. To eliminate outliers, the Isolation Forest algorithm [Pedregosa2011] was employed prior to the computation.

Centroid of regional lung function parameters: The center of mass of each regional variable was computed to characterize the spatial distribution of a regional parameter in three dimensions (x, y, z), with respect to the center of the P5 regional grid, which were normalized to range from -100 to 100%.

**SUPPLEMENTAL TABLES**

**Supplemental Table 1.** List, definition and median value of global image-derived variables.

| Abbreviation | Definition | Unit | All Subjects | Non survivors | Survivors | p-value |
| --- | --- | --- | --- | --- | --- | --- |
| VL_P5_ | Total lung volume at 5 cmH2O | L | 2.37 [2.02, 2.96] | 2.41 [2.08, 3.13] | 2.33 [1.91, 2.71] | 0.4 |
| VL_P15_ | Total lung volume at 15 cmH2O | L | 2.76 [2.29, 3.29] | 2.78 [2.51, 3.81] | 2.68 [2.17, 3.21] | 0.3 |
| Vg_P5_ | Lung gas volume at 5 cmH2O | L | 0.69 [0.43, 1.10] | 0.68 [0.48, 0.99] | 0.83 [0.40, 1.22] | 0.8 |
| Vg_P15_ | Lung gas volume at 15 cmH2O | L | 1.14 [0.73, 1.67] | 1.12 [0.82, 1.54] | 1.14 [0.65, 1.73] | >0.9 |
| V_tissue-P5_ | Lung tissue volume at 5 cmH2O | L | 1.62 [1.38, 1.84] | 1.65 [1.41, 2.06] | 1.56 [1.36, 1.72] | 0.2 |
| V_tissue-P15_ | Lung tissue volume at 15 cmH2O | L | 1.62 [1.36, 1.86] | 1.67 [1.42, 2.02] | 1.57 [1.32, 1.73] | 0.12 |
| V_rec_ | Global recruited tissue volume 5 ® 15 cmH_2_O | L | 0.11 [0.07, 0.19] | 0.14 [0.09, 0.23] | 0.09 [0.07, 0.15] | 0.028 |
| V_hi_ | Global Hyperinflated tissue volume-15 cmH_2_O | L | 0.001 [0.000, 0.006] | 0.001 [0.000, 0.005] | 0.002 [0.000, 0.009] | >0.9 |
| V_rec_/V_hi_ | Global recruited/hyperinflated lung volume ratio |  | 0.99 [0.95, 1.00] | 0.99 [0.97, 1.00] | 0.99 [0.93, 1.00] | 0.6 |
| F_rec_ | Global recruited tissue volume/tissue volume-5 cmH_2_O |  | 0.07 [0.05, 0.11] | 0.08 [0.05, 0.12] | 0.07 [0.04, 0.10] | 0.11 |
| F_hi_ | Global hyperinflated tissue volume/tissue volume-5 cmH_2_O |  | 0.001 [0.000, 0.003] | 0.001 [0.000, 0.003] | 0.001 [0.000, 0.005] | 0.9 |

**Supplemental Table 1.** See text for the detailed definition of the variables. HU: Hounsfield units. Variables without units are dimensionless.

**Supplemental Table 2.** List, definition and median value of regional image-derived variables.

| Abbreviation | Definition | Unit | All Subjects | Non survivors | Survivors | p-value |
| --- | --- | --- | --- | --- | --- | --- |
| ΔHU m | Regional density change, mean | HU | 70 [47, 93] | 75 [48, 93] | 68 [45, 90] | 0.4 |
| ΔHU sd | Regional density change, SD | HU | 76 [61, 86] | 75 [61, 88] | 77 [62, 85] | >0.9 |
| ΔHU med | Regional density change, median | HU | 55 [27, 79] | 61 [36, 82] | 55 [24, 74] | 0.3 |
| ΔHU iqr | Regional density change, IQR | HU | 90 [64, 114] | 90 [71, 120] | 88 [57, 110] | 0.4 |
| ΔHU kurt | Regional density change, kurtosis |  | 1.16 [0.49, 2.21] | 1.12 [0.41, 1.80] | 1.25 [0.51, 2.36] | 0.2 |
| ΔHU skew | Regional density change, skewness |  | 0.97 [0.67, 1.22] | 0.93 [0.64, 1.22] | 0.99 [0.75, 1.23] | 0.4 |
| ΔHU x | ΔHU, center of mass x relative distance from grid origin | % | -2 [-7, 5] | -2 [-8, 4] | -2 [-6, 5] | 0.5 |
| ΔHU y | ΔHU, center of mass y relative distance from grid origin | % | -20 [-26, -14] | -20 [-25, -13] | -20 [-26, -14] | 0.9 |
| ΔHU z | ΔHU, center of mass z relative distance from grid origin | % | 7 [0, 12] | 7 [-1, 14] | 7 [1, 12] | 0.9 |
| ΔV m | Regional gas volume change/region volume-5 cmH_2_O, mean |  | 0.16 [0.11, 0.19] | 0.16 [0.11, 0.19] | 0.17 [0.11, 0.20] | >0.9 |
| ΔV sd | Regional gas volume change/region volume-5 cmH_2_O, SD |  | 0.16 [0.13, 0.18] | 0.16 [0.13, 0.18] | 0.16 [0.13, 0.18] | 0.7 |
| ΔV med | Regional gas volume change/region volume-5 cmH_2_O, median |  | 0.12 [0.04, 0.16] | 0.11 [0.07, 0.16] | 0.12 [0.02, 0.17] | >0.9 |
| ΔV iqr | Regional gas volume change/region volume-5 cmH_2_O, IQR |  | 0.22 [0.17, 0.26] | 0.20 [0.17, 0.26] | 0.22 [0.18, 0.25] | >0.9 |
| ΔV kurt | Regional gas volume change/region volume-5 cmH_2_O, kurtosis |  | 0.55 [0.07, 1.39] | 0.75 [0.03, 1.44] | 0.45 [0.10, 1.37] | 0.8 |
| ΔV skew | Regional gas volume change/region volume-5 cmH_2_O, skewness |  | 0.91 [0.73, 1.35] | 0.90 [0.71, 1.34] | 0.92 [0.73, 1.36] | 0.9 |
| ΔV x | ΔV, center of mass x relative distance from grid origin | % | -3 [-13, 3] | -5 [-15, 1] | 0 [-9, 4] | 0.077 |
| ΔV y | ΔV, center of mass y relative distance from grid origin | % | -4 [-11, 3] | -5 [-13, 0] | -3 [-9, 7] | 0.060 |
| ΔV z | ΔV, center of mass z relative distance from grid origin | % | 16 [10, 23] | 13 [7, 22] | 18 [11, 25] | 0.056 |
| sΔV m | Specific volume change, mean |  | 1.11 [0.79, 2.01] | 1.05 [0.80, 1.92] | 1.31 [0.76, 2.08] | 0.7 |
| sΔV sd | Specific volume change, SD |  | 2.9 [1.7, 5.5] | 2.6 [1.7, 4.7] | 3.4 [1.7, 6.5] | 0.4 |
| sΔV med | Specific volume change, median |  | 0.48 [0.32, 0.64] | 0.51 [0.33, 0.66] | 0.44 [0.31, 0.60] | 0.3 |
| sΔV iqr | Specific volume change, IQR |  | 0.96 [0.64, 1.47] | 0.99 [0.68, 1.55] | 0.86 [0.63, 1.42] | 0.5 |
| sΔV kurt | Specific volume change, kurtosis |  | 41 [29, 50] | 40 [29, 50] | 42 [29, 51] | 0.7 |
| sΔV skew | Specific volume change, skewness |  | 5.62 [4.67, 6.26] | 5.50 [4.74, 6.23] | 5.81 [4.66, 6.37] | 0.5 |
| sΔV x | Specific volume change, center of mass x relative distance from grid origin | % | -3 [-11, 3] | -5 [-15, 0] | -1 [-7, 4] | 0.10 |
| sΔV y | Specific volume change, center of mass y relative distance from grid origin | % | -7 [-14, 0] | -9 [-15, -3] | -7 [-13, 6] | 0.12 |
| sΔV z | Specific volume change, center of mass z relative distance from grid origin | % | 15 [8, 23] | 10 [6, 22] | 17 [10, 24] | 0.050 |
| J m | Jacobian determinant, mean |  | 1.15 [1.09, 1.18] | 1.15 [1.09, 1.18] | 1.15 [1.10, 1.19] | >0.9 |
| J sd | Jacobian determinant, SD |  | 0.21 [0.19, 0.24] | 0.21 [0.19, 0.24] | 0.21 [0.19, 0.24] | 0.7 |
| J med | Jacobian determinant, median |  | 1.13 [1.06, 1.17] | 1.13 [1.08, 1.17] | 1.13 [1.06, 1.17] | 0.8 |
| J iqr | Jacobian determinant, IQR |  | 0.27 [0.24, 0.30] | 0.27 [0.23, 0.31] | 0.27 [0.25, 0.30] | 0.8 |
| J kurt | Jacobian determinant, kurtosis |  | 0.49 [0.29, 0.69] | 0.52 [0.30, 0.76] | 0.48 [0.28, 0.66] | 0.4 |
| J skew | Jacobian determinant, skewness |  | 0.35 [0.25, 0.47] | 0.35 [0.25, 0.48] | 0.35 [0.27, 0.45] | >0.9 |
| J x | Jacobian determinant, center of mass x relative distance from grid origin | % | -4.1 [-7.0, -0.6] | -4.6 [-7.5, -1.4] | -3.6 [-5.8, 0.1] | 0.2 |
| J y | Jacobian determinant, center of mass y relative distance from grid origin | % | -11.7 [-14.7, -6.2] | -11.8 [-14.7, -7.0] | -11.7 [-14.6, -6.0] | 0.9 |
| J z | Jacobian determinant, center of mass z relative distance from grid origin | % | 8.5 [4.9, 11.9] | 8.2 [4.0, 12.1] | 8.5 [6.0, 11.8] | 0.9 |
| Rec m | Regional recruited /regional tissue mass-5 cmH_2_O, mean |  | 0.06 [0.03, 0.08] | 0.06 [0.04, 0.10] | 0.05 [0.03, 0.07] | 0.2 |
| Rec sd | Regional recruited /regional tissue mass -5 cmH_2_O, SD |  | 0.20 [0.15, 0.23] | 0.20 [0.15, 0.24] | 0.19 [0.16, 0.22] | 0.9 |
| Rec med | Regional recruited /regional tissue mass -5 cmH_2_O, median |  | 0.000 [0.000, 0.007] | 0.000 [0.000, 0.009] | 0.000 [0.000, 0.001] | 0.4 |
| Rec iqr | Regional recruited /regional tissue mass -5 cmH_2_O, IQR |  | 0.12 [0.06, 0.21] | 0.14 [0.08, 0.22] | 0.09 [0.06, 0.19] | 0.3 |
| Rec kurt | Regional recruited /regional tissue mass -5 cmH_2_O, kurtosis |  | 3.8 [1.7, 5.4] | 3.3 [1.7, 5.4] | 4.4 [1.9, 5.4] | 0.4 |
| Rec skew | Regional recruited /regional tissue mass -5 cmH_2_O, skewness |  | 1.19 [0.49, 1.68] | 1.15 [0.53, 1.63] | 1.22 [0.44, 1.72] | 0.8 |
| Rec x | Rec, center of mass x relative distance from grid origin | % | -1 [-10, 4] | -1 [-12, 3] | -2 [-8, 5] | 0.6 |
| Rec y | Rec, center of mass y relative distance from grid origin | % | -22 [-29, -13] | -22 [-29, -15] | -22 [-29, -10] | 0.5 |
| Rec z | Rec, center of mass z relative distance from grid origin | % | 9 [0, 17] | 10 [-4, 17] | 9 [1, 17] | 0.6 |
| HI m | Regional hyperinflated /regional tissue mass-5 cmH_2_O, mean |  | 0.001 [0.000, 0.006] | 0.001 [0.000, 0.005] | 0.001 [0.000, 0.013] | 0.7 |
| HI sd | Regional hyperinflated /regional tissue mass-5 cmH_2_O, hyperinflation, SD |  | 0.01 [0.00, 0.03] | 0.01 [0.00, 0.03] | 0.01 [0.00, 0.06] | 0.9 |
| HI iqr | Regional hyperinflated /regional tissue mass-5 cmH_2_O, IQR |  | 0.000 [0.000, 0.000] | 0.000 [0.000, 0.000] | 0.000 [0.000, 0.000] | 0.8 |
| HI kurt | Regional hyperinflated /regional tissue mass-5 cmH_2_O, kurtosis |  | 111 [23, 6,010] | 404 [56, 9,336] | 73 [15, 2,471] | 0.3 |
| HI skew | Regional hyperinflated /regional tissue mass-5 cmH_2_O, skewness |  | 3 [-33, 5] | 0 [-64, 3] | 3 [2, 6] | 0.036 |
| HI x | HI, center of mass x relative distance from grid origin | % | -4 [-24, 5] | -1 [-26, 11] | -9 [-21, 0] | 0.6 |
| HI y | HI, center of mass y relative distance from grid origin | % | 14 [0, 28] | 15 [0, 28] | 12 [0, 27] | >0.9 |
| HI z | HI, center of mass z relative distance from grid origin | % | 9 [0, 21] | 9 [0, 22] | 9 [0, 17] | 0.6 |

**Supplemental Table 2.** See text for the detailed definition of the variables. HU: Hounsfield units. Variables without units are dimensionless.

**REFERENCES**

1. Dávila Serrano EE, Guigues L, Roux J-P, Cervenansky F, Camarasu-Pop S, Riveros Reyes JG, *et al.* CreaTools: A Framework to Develop Medical Image Processing Software: Application to Simulate Pipeline Stent Deployment in Intracranial Vessels with Aneurysms. In: Bolc L, Tadeusiewicz R, Chmielewski LJ, Wojciechowski K, editors. *Computer Vision and Graphics* Berlin, Heidelberg: Springer; 2012. p. 55–62.doi:10.1007/978-3-642-33564-8_7.

2. Klein S, Staring M, Murphy K, Viergever MA, Pluim JPW. elastix: A Toolbox for Intensity-Based Medical Image Registration. *IEEE Transactions on Medical Imaging* 2010;29:196–205.

3. Heinrich MP, Jenkinson M, Brady M, Schnabel JA. MRF-based deformable registration and ventilation estimation of lung CT. *IEEE transactions on medical imaging* 2013;32:1239–1248.

4. Shekarnabi MM. CT Registration-Derived Lung Function Imaging. 2023;at <https://theses.hal.science/tel-04507177>.

5. Simon BA. Non-invasive imaging of regional lung function using x-ray computed tomography. *Journal of clinical monitoring and computing* 2000;16:433–442.

6. Gattinoni L, Chiumello D, Caironi P, Busana M, Romitti F, Brazzi L, *et al.* COVID-19 pneumonia: different respiratory treatments for different phenotypes? *Intensive Care Med* 2020;46:1099–1102.
